# Internalized GPCRs as Potential Therapeutic Targets for the Management of Pain

**DOI:** 10.3389/fnmol.2019.00273

**Published:** 2019-11-12

**Authors:** Jeffri S. Retamal, Paulina D. Ramírez-García, Priyank A. Shenoy, Daniel P. Poole, Nicholas A. Veldhuis

**Affiliations:** ^1^Drug Discovery Biology Theme, Monash Institute of Pharmaceutical Sciences, Monash University, Parkville, VIC, Australia; ^2^Australian Research Council Centre of Excellence in Convergent Bio-Nano Science and Technology, Monash University, Parkville, VIC, Australia; ^3^Department of Anatomy and Neuroscience, The University of Melbourne, Parkville, VIC, Australia

**Keywords:** pain, analgesia, GPCR, trafficking, endosome, drug delivery, signal transduction

## Abstract

Peripheral and central neurons in the pain pathway are well equipped to detect and respond to extracellular stimuli such as pro-inflammatory mediators and neurotransmitters through the cell surface expression of receptors that can mediate rapid intracellular signaling. Following injury or infection, activation of cell surface G protein-coupled receptors (GPCRs) initiates cell signaling processes that lead to the generation of action potentials in neurons or inflammatory responses such as cytokine secretion by immune cells. However, it is now appreciated that cell surface events alone may not be sufficient for all receptors to generate their complete signaling repertoire. Following an initial wave of signaling at the cell surface, active GPCRs can engage with endocytic proteins such as the adaptor protein β-arrestin (βArr) to promote clathrin-mediated internalization. Classically, βArr-mediated internalization of GPCRs was hypothesized to terminate signaling, yet for multiple GPCRs known to contribute to pain, it has been demonstrated that endocytosis can also promote a unique “second wave” of signaling from intracellular membranes, including those of endosomes and the Golgi, that is spatiotemporally distinct from initial cell-surface events. In the context of pain, understanding the cellular and molecular mechanisms that drive spatiotemporal signaling of GPCRs is invaluable for understanding how pain occurs and persists, and how current analgesics achieve efficacy or promote side-effects. This review article discusses the importance of receptor localization for signaling outcomes of pro- and anti-nociceptive GPCRs, and new analgesic opportunities emerging through the development of “location-biased” ligands that favor binding with intracellular GPCR populations.

## Introduction

The sensation and transmission of pain are essential physiological processes that allow us to detect and react to harmful stimuli and initiate inflammatory responses to protect damaged tissue and promote wound healing. Peripheral and central processes that lead to pain transmission are highly adaptive, and the pain experienced is usually proportional to the extent of the injury. As a part of this adaptive physiological response, a heightened sensitivity to pain occurs to provide awareness of damaged tissue and maintain protective behavior for the duration of an injury.

As healing occurs, this sensitization typically reduces over time. In contrast, in chronic inflammatory and neuropathic pain conditions such as arthritis, fibromyalgia or diabetic-related neuropathy, where damaged tissue is unable to heal or inflammatory mediators continue to be produced, this sensitization fails to diminish and can cause significant discomfort and loss of function over extended time periods (Scholz and Woolf, 2002). This is typically described through two phenomena: (a) *allodynia*, where one feels pain in response to a normally non-painful stimulus; and (b) *hyperalgesia*, where one experiences an exacerbated pain sensation to a moderately painful stimulus (Baron, 2006; Steeds, 2016). Due to the complexity of chronic pain and significant limitations with safety and compliance for available analgesics, these conditions are extremely difficult to manage, thus impacting the quality of life for many patients.

Despite many advances in basic research and in the clinic, the analgesic landscape in recent decades has seen few changes, due to the limited availability of effective analgesic agents and the potential for abuse of routinely prescribed drugs (Dowell et al., 2016; Goodman and Brett, 2017). In the midst of a growing opioid crisis (Schuchat et al., 2017), the development of new pain medicines is becoming increasingly important. For safety and logistical reasons, the most obvious gains can be made by repurposing Food and Drug Administration (FDA)-approved drugs that are currently used for other indications (e.g., anti-depressants; Kremer et al., 2016; Cooper et al., 2017) or re-formulating established analgesics such as opioids to improve pharmacokinetic profiles (Saraghi and Hersh, 2013). However, new and effective therapeutic approaches may also be gained through greater characterization of the underlying cellular and molecular mechanisms that lead to pain, as a means to identify new molecular targets and further define how analgesic side-effects occur and can be avoided.

G protein-coupled receptors (GPCRs) are important mediators of pain or analgesia and many of these receptors participate in dynamic trafficking processes such as endocytosis, as a part of their activity cycle. It is now evident that receptor trafficking is also critical for the initiation of spatially and temporally distinct signaling events, and importantly, some of these location-specific or compartmentalized processes are associated with greater modulation of pain (Geppetti et al., 2015; Irannejad et al., 2017; Stoeber et al., 2018; Thomsen et al., 2018). Here, we address limitations of the current analgesic landscape and look to new drug discovery studies focused on GPCRs that participate in dynamic trafficking processes in neurons. New biophysical tools that have been used to characterize compartmentalized signaling reveal how the membrane partitioning properties of drugs influence their functional selectivity for location-specific processes. This knowledge has been exploited through the use of lipid-anchored drug conjugates that increase GPCR targeting in specific subcellular domains, to enhance analgesic outcomes through the inhibition of endosomal signaling.

## Challenges and Limitations of Current Analgesics

Chronic or persistent pain incorporates a complex range of disorders that requires a combination of non-pharmacological and pharmacological approaches for treatment. From a pharmacological perspective, treatment is possible by administering one or more therapeutic agents such as paracetamol/acetaminophen, non-steroidal anti-inflammatory drugs (NSAIDs) or cyclooxygenase-2 inhibitors (Coxibs) followed by careful use of opioids for elevated pain (e.g., morphine or oxycodone). Unfortunately, each of these drugs has associated side-effects that limit their use. NSAIDs and Coxibs have potential cardiovascular and gastrointestinal side effects (Whelton, 2000), and should be used more sparingly than paracetamol/acetaminophen, which carries a risk of hepatotoxicity with excessive use (Mahadevan et al., 2006). While opioids remain some of the most effective analgesics available in the clinic, they have a high abuse potential due to their euphoric or addictive properties, and where repeated use leads to receptor desensitization and tolerance. To overcome tolerance, patients with chronic pain can be subjected to sustained increases in dosing or switching to other more potent opioids to improve analgesia, which often provides only temporary gains in pain relief. However, this approach may increase the risk of tolerance and addiction over time, in addition to increasing the likelihood of debilitating side-effects such as constipation and respiratory depression (Corbett et al., 2006; Boudreau et al., 2009; Volkow et al., 2011).

Alternative GPCR targets have been identified to reduce reliance on opioid analgesics. Cannabinoids, which are proposed as effective opioid alternatives, reduce pain through activation of G_i/o_-coupled cannabinoid receptors (primarily CB_1_), which leads to the downregulation of excitatory processes, and modulation of serotoninergic (5-HT) and noradrenergic pathways. Although widely available and used for millennia, we are yet to see the outcomes of systematic use in the clinic for treating pain, and it is also acknowledged to lead to behavioral risks that require further investigation (Mendiguren et al., 2018). Gabapentinoids such as gabapentin or pregabalin, target the α2δ subunit of voltage-gated calcium channels and have been approved as first-line medications to manage neuropathic pain (e.g., postherpetic neuralgia, fibromyalgia). These were initially used for the treatment of epilepsy, and in some cases for anxiety disorders. Although regarded as relatively safe drugs, safety concerns for gabapentinoids have grown and include excessive usage and behavioral risks such as suicidal behavior (Johansen, 2018; Molero et al., 2019). Together, this provides a small insight into established and emerging risks associated with common analgesics. This raises the question of whether any of these compounds can be modified to improve their safety profiles and if new or emerging targets are available. We discuss these points below in the context of receptor trafficking, which is a critical component of the activity cycle for many molecular pain targets.

## Targeting GPCRs for The Treatment of Pain

Members of the GPCR superfamily are considered to be druggable targets due to high levels of cell surface expression and their ability to contribute to all pathophysiological processes, including pain. Accordingly, GPCR-selective drugs represent more than one-third of all FDA-approved medicines (Hauser et al., 2017). There are at least 40 members of the GPCR family that are considered to be potential therapeutic targets for the regulation of pain (Stone and Molliver, 2009). Yet despite advanced drug discovery programs for multiple receptors, and abuse concerns for opioid receptors, very few targets have clinically succeeded beyond opioids in the past decade, with notable exceptions being the recent approval of Fremanezumab, Eptinezumab, Galcanezumab, and Erenumab for treatment of migraine, being monoclonal antibodies that target the neuropeptide calcitonin gene-related peptide (CGRP) or its receptor, Calcitonin Receptor-Like Receptor/Receptor Activity-Modifying Protein 1 (CLR/RAMP1; see review by Scuteri et al., 2019).

**Figure 1:**
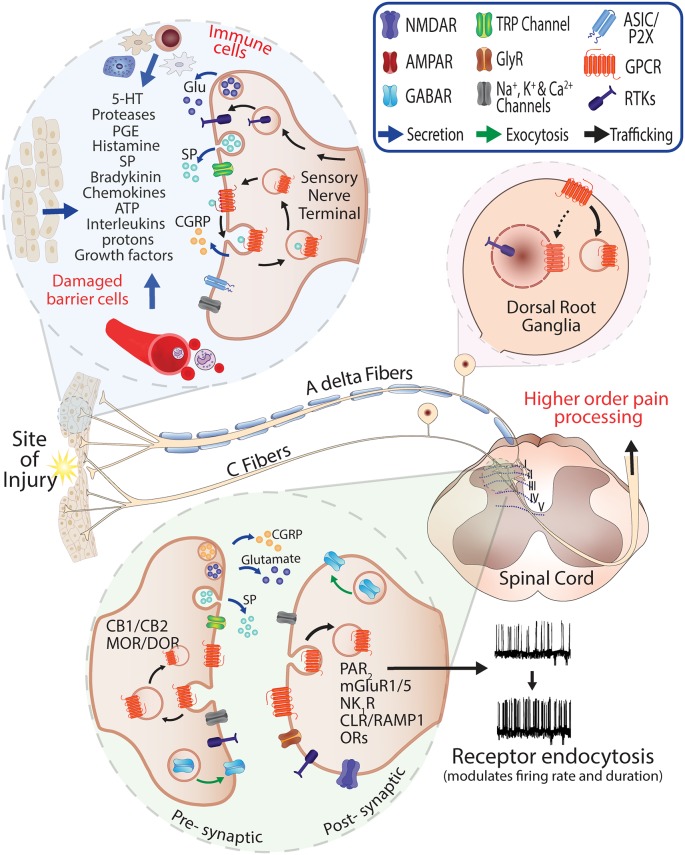
Role of G protein-coupled receptors (GPCRs) in pain and neurogenic inflammation. Injury or damaged tissues and infiltrating immune cells stimulate GPCRs on the peripheral sensory nerve terminals through release of painful and inflammatory mediators. Activated peptidergic and non-peptidergic Aδ and C fibers contribute to the response *via* the release of glutamate, Substance P (SP) and Calcitonin Gene-Related Peptide (CGRP) at the injury site and central terminals. The presence of endogenous mediators in the spinal cord (neuro- and glio-transmitters) can promote activation and recycling of GPCRs including pre-synaptic CB_1/2_ cannabinoid and Mu-opioid receptor (MOR)/Delta-opioid receptor (DOR) and the exocytic trafficking of the γ-aminobutyric acid_A_ receptor (GABAR) which is an inhibitory receptor that can normalize neuronal excitability where excitatory neurotransmitters are released. Stimulation and endocytosis of receptors such as Neurokinin 1 Receptor (NK_1_R) and Calcitonin Receptor-Like Receptor/Receptor Activity-Modifying Protein 1 (CLR/RAMP1) in post-synaptic neurons are known to modify firing frequency and the duration of pain responses (Jensen et al., 2017; Yarwood et al., 2017; Stoeber et al., 2018).

There are a number of challenges in the early phase of analgesic drug discovery for GPCRs. This includes safety concerns for targets that have overlapping functions in other tissues, and inaccurate evaluations of efficacy when using relatively simplified rodent-based pre-clinical pain models to represent the complexity of clinical pain conditions or characterize human-selective compounds (Mao, 2012). Furthermore, the localization of receptors in pre-synaptic and post-synaptic neurons is critical for the activity cycle and nociceptive outputs of several GPCRs (Figure 1). On a cellular level, considerations for the intracellular disposition of analgesics and their ability to regulate receptor trafficking and localization have also recently been proposed to be an important part of the drug discovery process (Jensen et al., 2017; Yarwood et al., 2017; Jimenez-Vargas et al., 2018; Stoeber et al., 2018).

## Receptor Trafficking Leads to Spatiotemporally Distinct Signaling Processes

GPCRs are highly dynamic proteins that achieve distinct signaling outcomes by adopting different conformational states (Rasmussen et al., 2011; Latorraca et al., 2017). Extracellular ligands that bind cell surface GPCRs promote receptor conformations that activate heterotrimeric G proteins to transduce downstream signaling and also favor phosphorylation by GPCR kinases (GRKs). This phosphorylation occurs primarily at the C-terminus to enhance engagement with β-arrestins (βArrs), which can function as adaptor proteins to mediate distinct signaling processes such as MAPK activity, and also facilitate interactions with clathrin-coated membranes to promote endocytosis into endosomes (Ferguson et al., 1996). This was historically considered to facilitate termination of signaling by targeting receptors to degradative pathways, or rapid receptor recycling to reset the activity cycle during the internalization process, and increase the potential for sustained signaling once the receptor is recovered at the plasma membrane (PM; Ferguson et al., 1996; Shukla et al., 2014).

A more recent theory has emerged, suggesting that a third trafficking possibility exists, whereby receptors can remain on intracellular membranes such as endosomes for sustained periods of time, to facilitate distinct signaling processes in a βArr- or a G protein-dependent manner. This paradigm shift was initially revealed by studies on Gs-coupled receptors such as the parathyroid (PTHR), thyroid-stimulating hormone (TSHR) and β2 adrenergic receptors to demonstrate that endosomal-mediated sustained cyclic adenosine monophosphate (cAMP) production could be observed after endocytosis has occurred (reviewed in detail by Vilardaga et al., 2014; Tsvetanova et al., 2015; Thomsen et al., 2018).

The development of genetically encoded tools such as conformation-selective nanobodies, Förster/Fluorescence Resonance Energy Transfer (FRET) or Bioluminescence Resonance Energy Transfer (BRET) biosensors, have provide highly sensitive approaches for observing and measuring dynamic activation states and spatiotemporal signaling [e.g., compartmentalized cAMP production, Protein kinase C (PKC) and Extracellular signal-regulated kinases (ERK) activity] of GPCRs in real-time (Irannejad et al., 2017; Halls and Canals, 2018). Given the prevalence and importance of trafficking GPCRs in neurons, the internalization and location-specific signaling of several GPCRs with established roles in pain have been described, including but not limited to the Neurokinin 1 Receptor (NK_1_R), CLR/RAMP1, metabotropic glutamate receptor 5 (mGluR5), chemokine receptor (CCR1), Protease-Activated Receptor 2 (PAR_2_) and the Mu Opioid Receptor (MOR; Mantyh et al., 1995a; O’Malley et al., 2003; Gilliland et al., 2013; Poole et al., 2015; Jensen et al., 2017; Yarwood et al., 2017; Stoeber et al., 2018). An overview of these trafficking outcomes is summarized in Table 1, to reveal how stimulation with endogenous ligands alters receptor localization *in vitro*, or in pre-clinical pain models.

**Table 1:** Receptors in pain pathways that undergo stimulation-induced endocytosis.

Receptor family	Endogenous stimuli	Localization (unstimulated)	Pain/Stimulus-induced trafficking	Reference
Mu and Delta Opioid Receptors (MOR, DOR)	Enkephalins Dynorphins	PM TGN	PM → Endosomes Direct activation on TGN by morphine	Sternini et al. (1996), Haberstock-Debic et al. (2005) and Stoeber et al. (2018)
Endocannabinoid Receptors (CB1, CB2)	AEA 2-AG	PM	PM, Endosomes	Rozenfeld and Devi (2008), Lever et al. (2009) and Flores-Otero et al. (2014)
Metabotropic Glutamate Receptor 5 (mGluR5)	Glutamate	PM ER Nucleus	PM Direct activation on Nuclear inner membrane	O’Malley et al. (2003) and Vincent et al. (2016), Vincent et al. (2017)
Protease-Activated Receptor 2 (PAR_2_)	Trypsin, Tryptase, Elastase, Cathepsin S	PM TGN	PM → Endosomes PM → Lysosomes	DeFea et al. (2000), Ricks and Trejo (2009) and Jimenez-Vargas et al. (2018)
Neurokinin 1 Receptor (NK_1_R)	Substance P Neurokinin A/B	PM	PM → Endosomes	Mantyh et al. (1995a,b) and Jensen et al. (2017)
Calcitonin Receptor-Like Receptor; Receptor Activity-Modifying Protein 1 (CLR/RAMP1)	CGRP Amylin	PM	PM → Endosomes	Padilla et al. (2007) and Yarwood et al. (2017)
Angiotensin Receptor 1 (AT_1_R)	Angiotensin II	PM	PM → Endosomes	Hein et al. (1997)
5-Hydroxytryptamine Receptor (5-HT2A)	Serotonin	PM	PM → Endosomes	Bhattacharyya et al. (2002) and Freeman et al. (2006)

*PM, plasma membrane; ER, endoplasmic reticulum, TGN, *trans*-Golgi Network; → denotes direction of receptor trafficking, from unstimulated receptor location to stimulated receptor location*.

## Ligands Exert Location Biased Effects by Accessing Different Receptor Pools

More recently, conformation-selective single-domain camelid antibodies (nanobodies) that can detect and bind active-state receptors have been instrumental for advancing this concept to other organelles. Distinct nanobody clones that are known to engage with the β_1_ Adrenergic Receptor (β_1_AR) or MOR have been shown to be recruited to the Golgi apparatus in a GPCR activity-dependent manner independently from initial stimulation at the cell surface. Specifically, this is achieved using relatively lipophilic ligands that can freely diffuse throughout the cell, or hydrophilic compounds that are proposed to access Golgi pools *via* transporters (Irannejad et al., 2017; Stoeber et al., 2018).

These important pharmacological insights have significant implications for understanding how drugs may exert their effects (or side-effects) and are consistent with other receptors that contribute to pain transmission. For example, endogenous peptide-based enkephalins can stimulate MOR and Delta-Opioid Receptor (DOR) to activate rapid signaling processes in micro-domains of the cell surface and sustained signaling from endosomes (Finn and Whistler, 2001; Groer et al., 2011; Halls et al., 2016; Stoeber et al., 2018), whereas non-peptide opioids such as morphine can freely diffuse through cells to stimulate Golgi pools of the MOR, and initiate a spatiotemporally distinct wave of signaling. The importance of opioid-induced Golgi signaling for analgesia and its association with safety outcomes remains to be determined *in vivo* (Stoeber et al., 2018).

Under pathological pain conditions, the excitatory mGluR5 has been detected in intracellular locations, including the inner nuclear membrane and endoplasmic reticulum (ER; Jong et al., 2014; Purgert et al., 2014; Vincent et al., 2016, 2017). Stimulated mGluR5 couples with Gα_q_ to evoke cytoplasmic and nuclear calcium mobilization (Jong et al., 2009). Furthermore, in models of spared-nerve injury (Vincent et al., 2016) and inflammatory pain (Vincent et al., 2017), 60% of the mGluR5 receptor population was shown to be localized to the inner nuclear membrane in spinal dorsal horn neurons (Vincent et al., 2016). Importantly, activation of nuclear mGluR5 leads to sustained nuclear Ca^2+^ signaling, phosphorylation of ERK1/2 and induction of c-fos expression, leading to increased nociceptive hypersensitivity (Lee et al., 2008; Jong et al., 2009; Purgert et al., 2014; Vincent et al., 2016, 2017). Blockade of cell surface mGluR5 by the impermeable antagonist LY393053 resulted in limited analgesia and modest reductions in second messenger coupling. In contrast, the membrane-permeable antagonist fenobam significantly reduced mechanical allodynia, MAP kinase (ERK1/2) phosphorylation and c-fos expression in a spared-nerve injury pain model. Although these differences may be caused by a range of factors including drug disposition and differences in potencies, it may also provide indirect evidence for the initiation of distinct mGluR5-dependent pain responses from different cellular locations (Lax et al., 2014; Vincent et al., 2016, 2017). Focused drug discovery around cell-permeant compounds biased toward intracellular mGluR5 pools is warranted and may lead to new opportunities for targeting glutamate signaling for analgesia.

## Modifying Intrinsic Drug Properties to Influence Location Bias

The studies above suggest that GPCRs that undergo endocytosis may be modulated more effectively by ligands that can diffuse to intracellular sites. This raises questions about whether the intrinsic properties of analgesic agents can be enhanced by chemical modification, to increase activity or partitioning into membranes where GPCRs are known to initiate signals associated with pain.

### Lipid-Anchored Ligands for Increased Endosomal Accumulation

The NK_1_R, has an established role in pain transmission and is well known to internalize when stimulated by the neurotransmitter, Substance P (SP). Peripheral inflammation-induced either acutely with intraplantar capsaicin or over sustained periods with Complete Freund’s Adjuvant, leads to pre-synaptic release of SP from primary afferent terminals onto the dorsal horn, and evokes robust NK_1_R internalization in Lamina I and II neurons of the spinal cord (Mantyh et al., 1995a; Abbadie et al., 1996, 1997; Jensen et al., 2017). Analogous to the endosomal signaling phenomena described above, it has also recently been reported that NK_1_R can mediate compartmentalized signaling processes including sustained PKC, nuclear ERK activity and cAMP production, in a clathrin/dynamin and βArr-dependent manner (Jensen et al., 2014, 2017; Poole et al., 2015). Similarly, CLR/RAMP1 which has an established role in central pain transmission and migraine pain (Lee and Kim, 2007; Bell, 2014), can undergo a CGRP-mediated redistribution into endosomes in HEK cells (Padilla et al., 2007) and in spinal cord sections (Yarwood et al., 2017). *In vitro* studies to clarify CLR/RAMP1-mediated compartmentalized signaling also showed that endocytosed receptor is associated with sustained nuclear ERK activity, cytosolic PKC activity and cytosolic cAMP production in HEK cells, and mediates sustained neuronal excitation in electrophysiological studies on rat spinal cord slices (Yarwood et al., 2017).

To demonstrate a similar potential for targeting endosomal receptor populations in peripheral neurons, PAR_2_ expressed on primary afferents is proposed to mediate inflammatory pain responses and its activity is strongly associated with irritable bowel syndrome (IBS). PAR_2_ signaling is also a stimulation-dependent process, where cleavage by different proteases can lead to distinct trafficking and location-based signaling outcomes. Trypsin proteolytically cleaves the extracellular amino terminus to activate PAR_2_ and promote PAR_2_ internalization into endosomes (DeFea et al., 2000; Ricks and Trejo, 2009). Endosomal PAR_2_ continues to signal through nuclear ERK and cytosolic PKC (Jimenez-Vargas et al., 2018). In contrast, elastase and cathepsin S mediated cleavage of the N-terminus activates PAR_2_ but does not stimulate PAR_2_ endocytosis (Zhao et al., 2014, 2015). Consequently, PM-delimited PAR_2_ signaling is relatively transient and is proposed to only mediate sustained signaling *via* activation of downstream effectors such as TRPV1 and TRPV4 ion channels (Poole et al., 2013; Jimenez-Vargas et al., 2018).

These data indicate that the internalization of excitatory GPCRs into endosomes may be associated with the generation of spatiotemporally distinct signaling profiles (Jensen et al., 2017; Yarwood et al., 2017; Jimenez-Vargas et al., 2018). Paradoxically, these internalized signaling processes are associated with persistent hyper-excitability of nociceptors and enhanced pain transmission through mechanisms that are not entirely clear, but require sustained kinase activity (Thomsen et al., 2018).

Pharmacological strategies have been employed to understand the importance of location bias of these receptors in pain transmission. Chemical modification by conjugation to the sterol cholestanol has previously been used by Simons and colleagues as a strategy to increase membrane affinity and the endosomal accumulation of a β-secretase transition state inhibitor (Rajendran et al., 2008). Using a similar lipid-anchor approach, antagonists for NK_1_R, CLR/RAMP1, and PAR_2_ were functionalized with the sterol moiety cholestanol, separated by a flexible polyethylene glycol (PEG_12_) linker. Focusing on the NK_1_R peptide antagonist spantide I (Jensen et al., 2017), the CLR/RAMP1 peptide antagonist CGRP_8–37_ (Yarwood et al., 2017) and I-343, a small molecule PAR_2_ antagonist (Jimenez-Vargas et al., 2018), the lipid anchor increased efficacy at the PM for all three compounds, and promoted incorporation and accumulation into endosomes, and is proposed to be maintained on the outer leaflet of membranes to target extracellular GPCR binding pockets, that are also accessible within the lumen of endosomes. This resulted in greater antagonism of endosomal-delimited signaling processes and more effective analgesia relative to unlipidated control compounds.

Alternative membrane-targeted antagonists have been developed for GPCRs, and the best studied of these are pepducins. Using peptides antagonists based on the sequences of GPCR intracellular domains to competitively bind G protein coupling, pepducins are anchored to membranes by chemical modification with palmitic acid (Covic et al., 2002), and these palmitoylated peptides have been proposed to flip to the inner leaflet of the PM to provide cell surface-delimited signaling inhibition. Pepducins are efficacious in inflammatory models (edema, osteoarthritis, sepsis) by selectively targeting GPCRs including PARs (PAR1, 2, 4) and chemokine receptors (CXCR1, 2, 4; Tressel et al., 2011; Tsuji et al., 2013).

**Figure 2:**
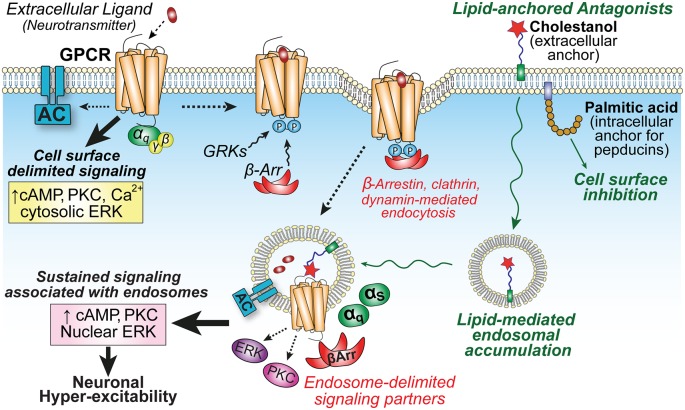
GPCR localization influences compartmentalized signaling and neuronal hyper-excitability. Activation of GPCRs on central neurons by extracellular neuropeptides (e.g., NK_1_R or CL/RAMP1) initiates cell surface-delimited G protein-dependent signaling events. This is followed by GPCR kinase (GRK) phosphorylation, arrestin-binding, and clathrin-mediated endocytosis into endosomes to promote the recruitment of unique signaling complexes and drive spatiotemporally distinct signaling that is associated with sustained excitability of neurons in spinal cord slice preparations (Jensen et al., 2017; Yarwood et al., 2017). Lipid conjugation can influence membrane partitioning of antagonists. Palmitoylated pepducins are proposed to inhibit G protein-mediated inflammatory processes on the cytoplasmic interface of the plasma membrane (PM); whereas the sterol moiety cholestanol increases drug accumulation in endosomes to enhance inhibition of sustained endosomal-delimited signaling.

Together, these studies support the use of lipid conjugation as a strategy for modifying the location biased profiles of drugs. The lipophilic properties of the anchor dominate the membrane partitioning of ligands, even hydrophobic small molecules, and are therefore a critical determinant for achieving unique membrane distributions, to improve ligand efficacy at specific subcellular locations (Figure 2). While pepducins have entered clinical trials (Gurbel et al., 2016), cholestanol conjugates that lead to the accumulation of ligands in endosomes have not advanced beyond pre-clinical pain models, but suggest that targeting endosomes through drug delivery strategies may be a useful therapeutic approach for the management of pathological pain.

### Modifying pH-Sensitivity of MOR-Opioid Interactions

Increasing ligand selectivity for GPCR binding under acidic conditions is a potential alternative strategy for favoring the modulation of GPCRs in endosomes. Relative to the physiological pH of the extracellular environment, trafficking proteins are exposed to an increasingly acidic gradient, as cargo is sorted deeper into the endosomal network. The reduction in pH increases proteolytic activity, which is essential for lysosomal protein degradation, and also for modulating the activity and presence of peptides such as SP or CGRP in endosomal compartments (Padilla et al., 2007).

With a need to reduce opioid-MOR interactions that lead to on-target side effects such as sedation, addiction and constipation, Stein and colleagues recently explored the potential for a pH-sensitive analog of the MOR agonist fentanyl to selectively engage with MOR only in pathological conditions, where acidosis is likely to occur (Spahn et al., 2017). The acid dissociation constant (pK_a_) of fentanyl is >8 and can activate MOR in physiological conditions (pH 7.4) and between pH 5 and 7, being the expected pH range within the microenvironment of inflamed tissue (Ludwig et al., 2003; Thurlkill et al., 2005). It was therefore hypothesized that reducing the pKa of fentanyl >7 by replacement of side-chain hydrogens would favor binding exclusively in pathological conditions.

Utilizing atomic-level structural information for MOR (Manglik et al., 2012) hydrogen replacement fentanyl analogs were designed and binding energies were measured in computational simulations, to identify candidates for further *in vitro* testing and assessment in pain models. The substitution of hydrogen by fluorine resulted in the development of (±)-N-(3-fluoro-1-phenethylpiperidine-4-yl)-N-phenyl propionamide (NFEPP) with a pK_a_ of 6.8 (Spahn et al., 2017). NFEPP and fentanyl were intravenously administered and compared using two models of persistent or acute inflammatory pain (Spahn et al., 2017) and more recently in neuropathic and abdominal pain in rats (Rodriguez-Gaztelumendi et al., 2018). Fentanyl produced analgesia in both injured and non-injured tissue. However, NFEPP analgesia was restricted to inflamed, acidic tissues. High doses of fentanyl induced respiratory depression, sedation and CNS-associated side-effects such as decrease of defecation, heart rate, and blood oxygen saturation, whereas NFEPP did not (Spahn et al., 2017; Rodriguez-Gaztelumendi et al., 2018).

These studies demonstrate the importance of protonation of ligands for receptor binding and activation, and the potential to modulate receptor affinity at pathological pH, thus limiting on-target side effects and unwanted MOR interactions in healthy tissues. The pH range of endosomes is comparable to inflamed tissue and hence, further *in vitro* studies may be useful to determine if the properties of NFEPP also enhance binding with endosomal receptor pools. Furthermore, if NFEPP maintains its ability to partition into membranes to access and activate the Golgi pool of MOR, this may suggest that MOR activation in the Golgi is favorable for analgesia, rather than being associated with poor safety outcomes.

## Concluding Remarks

The signaling and trafficking of GPCRs is important for mediating physiological processes at the PM and can also drive distinct, compartmentalized signaling events from intracellular sites. In the context of pain, defining this relationship may provide significant opportunities for neuropharmacology and analgesic drug discovery. However, while this may provide important insights that pinpoint discrete signaling outcomes most closely associated with modulating pain behaviors, or favorable drug properties that achieve analgesia while avoiding safety issues, it also critical to translate these proof of concept studies to human tissues and diseases. It remains unknown (and very challenging), for example, to demonstrate how the Golgi-specific MOR-signaling component influences analgesia or other side-effects in animals or humans, or if pH-sensitive fentanyl analogs provide genuine advantages over the parent compound in humans with chronic pain.

Although a relatively new phenomenon, ligands that have been identified or modified to possess unique location-biased properties have provided both interesting and valuable proof of concept findings that warrant further investigation. This includes receptors discussed in this review article and many others that contribute to pain in both neurons and non-neuronal cells that drive signaling processes that lead to sustained pain. With the availability of powerful new technologies and biophysical tools, it is predicted that further in-depth compartmentalized signaling-focused drug discovery studies on other trafficking receptors will provide many more valuable insights and other location-specific drug targets.

## Author Contributions

JR, PR-G, PS, DP and NV wrote the manuscript.

## Conflict of Interest

Research in NV and DP laboratories is funded in part by Takeda Pharmaceuticals Inc.

The remaining authors declare that the research was conducted in the absence of any commercial or financial relationships that could be construed as a potential conflict of interest.

## Abbreviations

GPCRs: G protein couple receptors
βArr: adaptor protein β-arrestin
FDA: Food and Drug Administration
NSAIDs: non-steroidal anti-inflammatory drugs
Coxibs: cyclooxygenase-2 inhibitors
CB1-2: Cannabinoid 1-2 receptors
SP: Substance P
NK_1_R: Neurokinin receptor 1
CGRP: Calcitonin gene-related peptide
CLR: Calcitonin receptor-like receptor
RAMP1: Receptor activity-modifying protein
MOR: Mu-opioid receptor
DOR: Delta-opioid receptor
mGluR5: Metabotropic glutamate 5 receptor
PAR_2_: Protease-activated receptor-2
5-HT: serotoninergic
CCR5: Chemokine receptor 5
GRKs: GPCR kinases
PTHR: parathyroid
TSHR: thyroid-stimulating hormone
β_1_AR: β_1_ Adrenergic
NFEPP: N-(3-fluoro-1-phenethylpiperidine-4-yl)-N-phenyl propionamide
ERK: Extracellular signal-regulated kinases
PKC: Protein kinase C
cAMP: Cyclic adenosine monophosphate
FRET: Resonance Energy Transfer
BRET: Bioluminescence Resonance Energy Transfer
ER: endoplasmic reticulum
TRPV1, TRPV4: Transient receptor potential cation channel subfamily V member 1-4.

## References

[B1] Abbadie C. Brown J. L. Mantyh P. W. Basbaum A. I. (1996). Spinal cord substance P receptor immunoreactivity increases in both inflammatory and nerve injury models of persistent pain. Neuroscience 70, 201–209. 10.1016/0306-4522(95)00343-h 8848125

[B2] Abbadie C. Trafton J. Liu H. Mantyh P. W. Basbaum A. I. (1997). Inflammation increases the distribution of dorsal horn neurons that internalize the neurokinin-1 receptor in response to noxious and non-noxious stimulation. J. Neurosci. 17, 8049–8060. 10.1523/JNEUROSCI.17-20-08049.1997 9315923 PMC6793895

[B3] Baron R. (2006). Mechanisms of disease: neuropathic pain-a clinical perspective. Nat. Clin. Pract. Neurol. 2, 95–106. 10.1038/ncpneuro0113 16932531

[B4] Bell I. M. (2014). Calcitonin gene-related peptide receptor antagonists: new therapeutic agents for migraine. J. Med. Chem. 57, 7838–7858. 10.1021/jm500364u 24960305

[B5] Bhattacharyya S. Puri S. Miledi R. Panicker M. M. (2002). Internalization and recycling of 5-HT2A receptors activated by serotonin and protein kinase C-mediated mechanisms. Proc. Natl. Acad. Sci. U S A 99, 14470–14475. 10.1073/pnas.212517999 12388782 PMC137907

[B6] Boudreau D. Von Korff M. Rutter C. M. Saunders K. Ray G. T. Sullivan M. D.. (2009). Trends in long-term opioid therapy for chronic non-cancer pain. Pharmacoepidemiol. Drug Saf. 18, 1166–1175. 10.1002/pds.1833 19718704 PMC3280087

[B7] Cooper T. E. Heathcote L. C. Clinch J. Gold J. I. Howard R. Lord S. M.. (2017). Antidepressants for chronic non-cancer pain in children and adolescents. Cochrane Database Syst. Rev. 8:CD012535. 10.1002/14651858.CD012535.pub2 28779487 PMC6424378

[B8] Corbett A. D. Henderson G. McKnight A. T. Paterson S. J. (2006). 75 years of opioid research: the exciting but vain quest for the Holy Grail. Br. J. Pharmacol. 147, S153–S162. 10.1038/sj.bjp.0706435 16402099 PMC1760732

[B9] Covic L. Gresser A. L. Talavera J. Swift S. Kuliopulos A. (2002). Activation and inhibition of G protein-coupled receptors by cell-penetrating membrane-tethered peptides. Proc. Natl. Acad. Sci. U S A 99, 643–648. 10.1073/pnas.022460899 11805322 PMC117359

[B10] DeFea K. A. Zalevsky J. Thoma M. S. Déry O. Mullins R. D. Bunnett N. W. (2000). β-arrestin-dependent endocytosis of proteinase-activated receptor 2 is required for intracellular targeting of activated ERK1/2. J. Cell Biol. 148, 1267–1281. 10.1083/jcb.148.6.1267 10725339 PMC2174299

[B11] Dowell D. Haegerich T. M. Chou R. (2016). CDC guideline for prescribing opioids for chronic pain-United States, 2016. JAMA 315, 1624–1645. 10.1001/jama.2016.1464 26977696 PMC6390846

[B12] Ferguson S. S. Downey W. E. Colapietro A. M. Barak L. S. Ménard L. Caron M. G. (1996). Role of β-arrestin in mediating agonist-promoted G protein-coupled receptor internalization. Science 271, 363–366. 10.1126/science.271.5247.363 8553074

[B13] Finn A. K. Whistler J. L. (2001). Endocytosis of the mu opioid receptor reduces tolerance and a cellular hallmark of opiate withdrawal. Neuron 32, 829–839. 10.1016/s0896-6273(01)00517-7 11738029

[B14] Flores-Otero J. Ahn K. H. Delgado-Peraza F. Mackie K. Kendall D. A. Yudowski G. A. (2014). Ligand-specific endocytic dwell times control functional selectivity of the cannabinoid receptor 1. Nat. Commun. 5:4589. 10.1038/ncomms5589 25081814 PMC4227836

[B15] Freeman S. L. Glatzle J. Robin C. S. Valdellon M. Sternini C. Sharp J. W.. (2006). Ligand-induced 5-HT3 receptor internalization in enteric neurons in rat ileum. Gastroenterology 131, 97–107. 10.1053/j.gastro.2006.04.013 16831594

[B16] Geppetti P. Veldhuis N. A. Lieu T. Bunnett N. W. (2015). G protein-coupled receptors: dynamic machines for signaling pain and itch. Neuron 88, 635–649. 10.1016/j.neuron.2015.11.001 26590341

[B17] Gilliland C. T. Salanga C. L. Kawamura T. Trejo J. Handel T. M. (2013). The chemokine receptor CCR1 is constitutively active, which leads to G protein-independent, β-arrestin-mediated internalization. J. Biol. Chem. 288, 32194–32210. 10.1074/jbc.m113.503797 24056371 PMC3820859

[B18] Goodman C. W. Brett A. S. (2017). Gabapentin and pregabalin for pain-is increased prescribing a cause for concern? N. Engl. J. Med. 377, 411–414. 10.1056/NEJMp1704633 28767350

[B19] Groer C. E. Schmid C. L. Jaeger A. M. Bohn L. M. (2011). Agonist-directed interactions with specific β-arrestins determine mu-opioid receptor trafficking, ubiquitination and dephosphorylation. J. Biol. Chem. 286, 31731–31741. 10.1074/jbc.M111.248310 21757712 PMC3173119

[B20] Gurbel P. A. Bliden K. P. Turner S. E. Tantry U. S. Gesheff M. G. Barr T. P.. (2016). Cell-penetrating pepducin therapy targeting PAR1 in subjects with coronary artery disease. Arterioscler. Thromb. Vasc. Biol. 36, 189–197. 10.1161/ATVBAHA.115.306777 26681756 PMC4836853

[B21] Haberstock-Debic H. Kim K.-A. Yu Y. J. von Zastrow M. (2005). Morphine promotes rapid, arrestin-dependent endocytosis of mu-opioid receptors in striatal neurons. J. Neurosci. 25, 7847–7857. 10.1523/JNEUROSCI.5045-04.2005 16120787 PMC6725258

[B22] Halls M. L. Canals M. (2018). Genetically encoded FRET biosensors to illuminate compartmentalised GPCR signalling. Trends Pharmacol. Sci. 39, 148–157. 10.1016/j.tips.2017.09.005 29054309

[B23] Halls M. L. Yeatman H. R. Nowell C. J. Thompson G. L. Gondin A. B. Civciristov S.. (2016). Plasma membrane localization of the μ-opioid receptor controls spatiotemporal signaling. Sci. Signal. 9:ra16. 10.1126/scisignal.aac9177 26861044

[B24] Hauser A. S. Attwood M. M. Rask-Andersen M. Schiöth H. B. Gloriam D. E. (2017). Trends in GPCR drug discovery: new agents, targets and indications. Nat. Rev. Drug Discov. 16, 829–842. 10.1038/nrd.2017.178 29075003 PMC6882681

[B25] Hein L. Meinel L. Pratt R. E. Dzau V. J. Kobilka B. K. (1997). Intracellular trafficking of angiotensin II and its AT1 and AT2 receptors: evidence for selective sorting of receptor and ligand. Mol. Endocrinol. 11, 1266–1277. 10.1210/mend.11.9.9975 9259318

[B26] Irannejad R. Pessino V. Mika D. Huang B. Wedegaertner P. B. Conti M.. (2017). Functional selectivity of GPCR-directed drug action through location bias. Nat. Chem. Biol. 13, 799–806. 10.1038/nchembio.2389 28553949 PMC5733145

[B27] Jensen D. D. Halls M. L. Murphy J. E. Canals M. Cattaruzza F. Poole D. P.. (2014). Endothelin-converting enzyme 1 and β-arrestins exert spatiotemporal control of substance P-induced inflammatory signals. J. Biol. Chem. 289, 20283–20294. 10.1074/jbc.M114.578179 24898255 PMC4106342

[B28] Jensen D. D. Lieu T. Halls M. L. Veldhuis N. A. Imlach W. L. Mai Q. N.. (2017). Neurokinin 1 receptor signaling in endosomes mediates sustained nociception and is a viable therapeutic target for prolonged pain relief. Sci. Transl. Med. 9:eaal3447. 10.1126/scitranslmed.aal3447 28566424 PMC6034632

[B29] Jimenez-Vargas N. N. Pattison L. A. Zhao P. Lieu T. Latorre R. Jensen D. D.. (2018). Protease-activated receptor-2 in endosomes signals persistent pain of irritable bowel syndrome. Proc. Natl. Acad. Sci. U S A 115, E7438–E7447. 10.1073/pnas.1721891115 30012612 PMC6077730

[B30] Johansen M. E. (2018). Gabapentinoid use in the United States 2002 through 2015. JAMA Intern. Med. 178, 292–294. 10.1001/jamainternmed.2017.7856 29297045 PMC5838608

[B31] Jong Y.-J. I. Kumar V. O’Malley K. L. (2009). Intracellular metabotropic glutamate receptor 5 (mGluR5) activates signaling cascades distinct from cell surface counterparts. J. Biol. Chem. 284, 35827–35838. 10.1074/jbc.M109.046276 19840937 PMC2791012

[B32] Jong Y.-J. I. Sergin I. Purgert C. A. O’Malley K. L. (2014). Location-dependent signaling of the group 1 metabotropic glutamate receptor mGlu5. Mol. Pharmacol. 86, 774–785. 10.1124/mol.114.094763 25326002 PMC4244594

[B33] Kremer M. Salvat E. Muller A. Yalcin I. Barrot M. (2016). Antidepressants and gabapentinoids in neuropathic pain: mechanistic insights. Neuroscience 338, 183–206. 10.1016/j.neuroscience.2016.06.057 27401055

[B34] Latorraca N. R. Venkatakrishnan A. J. Dror R. O. (2017). GPCR dynamics: structures in motion. Chem. Rev. 117, 139–155. 10.1021/acs.chemrev.6b00177 27622975

[B35] Lax N. C. George D. C. Ignatz C. Kolber B. J. (2014). The mGluR5 antagonist fenobam induces analgesic conditioned place preference in mice with spared nerve injury. PLoS One 9:e103524. 10.1371/journal.pone.0103524 25061818 PMC4111598

[B37] Lee S. E. Kim J.-H. (2007). Involvement of substance P and calcitonin gene-related peptide in development and maintenance of neuropathic pain from spinal nerve injury model of rat. Neurosci. Res. 58, 245–249. 10.1016/j.neures.2007.03.004 17428562

[B36] Lee J. H. Lee J. Choi K. Y. Hepp R. Lee J.-Y. Lim M. K.. (2008). Calmodulin dynamically regulates the trafficking of the metabotropic glutamate receptor mGluR5. Proc. Natl. Acad. Sci. U S A 105, 12575–12580. 10.1073/pnas.0712033105 18715999 PMC2527953

[B38] Lever I. J. Robinson M. Cibelli M. Paule C. Santha P. Yee L.. (2009). Localization of the endocannabinoid-degrading enzyme fatty acid amide hydrolase in rat dorsal root ganglion cells and its regulation after peripheral nerve injury. J. Neurosci. 29, 3766–3780. 10.1523/JNEUROSCI.4071-08.2009 19321773 PMC6665026

[B39] Ludwig M.-G. Vanek M. Guerini D. Gasser J. A. Jones C. E. Junker U.. (2003). Proton-sensing G-protein-coupled receptors. Nature 425, 93–98. 10.1038/nature01905 12955148

[B40] Mahadevan S. B. K. McKiernan P. J. Davies P. Kelly D. A. (2006). Paracetamol induced hepatotoxicity. Arch. Dis. Child. 91, 598–603. 10.1136/adc.2005.076836 16547087 PMC2082829

[B41] Manglik A. Kruse A. C. Kobilka T. S. Thian F. S. Mathiesen J. M. Sunahara R. K.. (2012). Crystal structure of the μ-opioid receptor bound to a morphinan antagonist. Nature 485, 321–326. 10.1038/nature10954 22437502 PMC3523197

[B42] Mantyh P. W. Allen C. J. Ghilardi J. R. Rogers S. D. Mantyh C. R. Liu H.. (1995a). Rapid endocytosis of a G protein-coupled receptor: substance P evoked internalization of its receptor in the rat striatum *in vivo*. Proc. Natl. Acad. Sci. U S A 92, 2622–2626. 10.1073/pnas.92.7.2622 7535928 PMC42270

[B43] Mantyh P. W. DeMaster E. Malhotra A. Ghilardi J. R. Rogers S. D. Mantyh C. R.. (1995b). Receptor endocytosis and dendrite reshaping in spinal neurons after somatosensory stimulation. Science 268, 1629–1632. 10.1126/science.7539937 7539937

[B44] Mao J. (2012). Current challenges in translational pain research. Trends Pharmacol. Sci. 33, 568–573. 10.1016/j.tips.2012.08.001 22959652 PMC3482290

[B45] Mendiguren A. Aostri E. Pineda J. (2018). Regulation of noradrenergic and serotonergic systems by cannabinoids: relevance to cannabinoid-induced effects. Life Sci. 192, 115–127. 10.1016/j.lfs.2017.11.029 29169951

[B46] Molero Y. Larsson H. D’Onofrio B. M. Sharp D. J. Fazel S. (2019). Associations between gabapentinoids and suicidal behaviour, unintentional overdoses, injuries, road traffic incidents and violent crime: population based cohort study in Sweden. BMJ 365:l2147. 10.1136/bmj.l2147 31189556 PMC6559335

[B47] O’Malley K. L. Jong Y.-J. I. Gonchar Y. Burkhalter A. Romano C. (2003). Activation of metabotropic glutamate receptor mGlu5 on nuclear membranes mediates intranuclear Ca^2+^ changes in heterologous cell types and neurons. J. Biol. Chem. 278, 28210–28219. 10.1074/jbc.M300792200 12736269

[B48] Padilla B. E. Cottrell G. S. Roosterman D. Pikios S. Muller L. Steinhoff M.. (2007). Endothelin-converting enzyme-1 regulates endosomal sorting of calcitonin receptor-like receptor and β-arrestins. J. Cell Biol. 179, 981–997. 10.1083/jcb.200704053 18039931 PMC2099187

[B49] Poole D. P. Amadesi S. Veldhuis N. A. Abogadie F. C. Lieu T. Darby W.. (2013). Protease-activated receptor 2 (PAR2) protein and transient receptor potential vanilloid 4 (TRPV4) protein coupling is required for sustained inflammatory signaling. J. Biol. Chem. 288, 5790–5802. 10.1074/jbc.M112.438184 23288842 PMC3581372

[B50] Poole D. P. Lieu T. Pelayo J. C. Eriksson E. M. Veldhuis N. A. Bunnett N. W. (2015). Inflammation-induced abnormalities in the subcellular localization and trafficking of the neurokinin 1 receptor in the enteric nervous system. Am. J. Physiol. Gastrointest. Liver Physiol. 309, G248–G259. 10.1152/ajpgi.00118.2015 26138465 PMC4537929

[B51] Purgert C. A. Izumi Y. Jong Y.-J. I. Kumar V. Zorumski C. F. O’Malley K. L. (2014). Intracellular mGluR5 can mediate synaptic plasticity in the hippocampus. J. Neurosci. 34, 4589–4598. 10.1523/JNEUROSCI.3451-13.2014 24672004 PMC3965784

[B52] Rajendran L. Schneider A. Schlechtingen G. Weidlich S. Ries J. Braxmeier T.. (2008). Efficient inhibition of the Alzheimer’s disease β-secretase by membrane targeting. Science 320, 520–523. 10.1126/science.1156609 18436784

[B53] Rasmussen S. G. F. Choi H.-J. Fung J. J. Pardon E. Casarosa P. Chae P. S.. (2011). Structure of a nanobody-stabilized active state of the β_2_ adrenoceptor. Nature 469, 175–180. 10.1038/nature09648 21228869 PMC3058308

[B54] Ricks T. K. Trejo J. (2009). Phosphorylation of protease-activated receptor-2 differentially regulates desensitization and internalization. J. Biol. Chem. 284, 34444–34457. 10.1074/jbc.M109.048942 19815543 PMC2797212

[B55] Rodriguez-Gaztelumendi A. Spahn V. Labuz D. Machelska H. Stein C. (2018). Analgesic effects of a novel pH-dependent μ-opioid receptor agonist in models of neuropathic and abdominal pain. Pain 159, 2277–2284. 10.1097/j.pain.0000000000001328 29994988 PMC6203420

[B56] Rozenfeld R. Devi L. A. (2008). Regulation of CB1 cannabinoid receptor trafficking by the adaptor protein AP-3. FASEB J. 22, 2311–2322. 10.1096/fj.07-102731 18267983 PMC3127579

[B57] Saraghi M. Hersh E. V. (2013). Three newly approved analgesics: an update. Anesth. Prog. 60, 178–187. 10.2344/0003-3006-60.4.178 24423420 PMC3891458

[B58] Scholz J. Woolf C. J. (2002). Can we conquer pain? Nat. Neurosci. 5, 1062–1067. 10.1038/nn942 12403987

[B59] Schuchat A. Houry D. Guy G. P. (2017). New data on opioid use and prescribing in the United States. JAMA 318, 425–426. 10.1001/jama.2017.8913 28687823 PMC5703201

[B60] Scuteri D. Adornetto A. Rombolà L. Naturale M. D. Morrone L. A. Bagetta G.. (2019). New trends in migraine pharmacology: targeting calcitonin gene-related peptide (CGRP) with monoclonal antibodies. Front. Pharmacol. 10:363. 10.3389/fphar.2019.00363 31024319 PMC6465320

[B61] Shukla A. K. Westfield G. H. Xiao K. Reis R. I. Huang L.-Y. Tripathi-Shukla P.. (2014). Visualization of arrestin recruitment by a G-protein-coupled receptor. Nature 512, 218–222. 10.1038/nature13430 25043026 PMC4134437

[B63] Spahn V. Del Vecchio G. Labuz D. Rodriguez-Gaztelumendi A. Massaly N. Temp J.. (2017). A nontoxic pain killer designed by modeling of pathological receptor conformations. Science 355, 966–969. 10.1126/science.aai8636 28254944

[B64] Steeds C. E. (2016). The anatomy and physiology of pain. Surgery 34, 55–59. 10.1016/j.mpsur.2015.11.005

[B65] Sternini C. Spann M. Anton B. Keith D. E. Bunnett N. W. von Zastrow M.. (1996). Agonist-selective endocytosis of mu opioid receptor by neurons *in vivo*. Proc. Natl. Acad. Sci. U S A 93, 9241–9246. 10.1073/pnas.93.17.9241 8799185 PMC38626

[B66] Stoeber M. Jullié D. Lobingier B. T. Laeremans T. Steyaert J. Schiller P. W.. (2018). A genetically encoded biosensor reveals location bias of opioid drug action. Neuron 98, 963.e5–976.e5. 10.1016/j.neuron.2018.04.021 29754753 PMC6481295

[B67] Stone L. S. Molliver D. C. (2009). In search of analgesia: emerging roles of GPCRs in pain. Mol. Interv. 9, 234–251. 10.1124/mi.9.5.7 19828831 PMC2861805

[B68] Thomsen A. R. B. Jensen D. D. Hicks G. A. Bunnett N. W. (2018). Therapeutic targeting of endosomal G-protein-coupled receptors. Trends Pharmacol. Sci. 39, 879–891. 10.1016/j.tips.2018.08.003 30180973 PMC6508874

[B69] Thurlkill R. L. Cross D. A. Scholtz J. M. Pace C. N. (2005). pKa of fentanyl varies with temperature: implications for acid-base management during extremes of body temperature. J. Cardiothorac. Vasc. Anesth. 19, 759–762. 10.1053/j.jvca.2004.11.039 16326301

[B70] Tressel S. L. Koukos G. Tchernychev B. Jacques S. L. Covic L. Kuliopulos A. (2011). Pharmacology, biodistribution and efficacy of GPCR-based pepducins in disease models. Methods Mol. Biol. 683, 259–275. 10.1007/978-1-60761-919-2_19 21053136 PMC3780409

[B71] Tsuji M. Ueda S. Hirayama T. Okuda K. Sakaguchi Y. Isono A.. (2013). FRET-based imaging of transbilayer movement of pepducin in living cells by novel intracellular bioreductively activatable fluorescent probes. Org. Biomol. Chem. 11, 3030–3037. 10.1039/c3ob27445d 23532512

[B72] Tsvetanova N. G. Irannejad R. von Zastrow M. (2015). G protein-coupled receptor (GPCR) signaling *via* heterotrimeric G proteins from endosomes. J. Biol. Chem. 290, 6689–6696. 10.1074/jbc.R114.617951 25605726 PMC4358092

[B73] Vilardaga J.-P. Jean-Alphonse F. G. Gardella T. J. (2014). Endosomal generation of cAMP in GPCR signaling. Nat. Chem. Biol. 10, 700–706. 10.1038/nchembio.1611 25271346 PMC4417940

[B74] Vincent K. Cornea V. M. Jong Y.-J. I. Laferrière A. Kumar N. Mickeviciute A.. (2016). Intracellular mGluR5 plays a critical role in neuropathic pain. Nat. Commun. 5:10604. 10.1038/ncomms10604 26837579 PMC4742982

[B75] Vincent K. Wang S. F. Laferrière A. Kumar N. Coderre T. J. (2017). Spinal intracellular metabotropic glutamate receptor 5 (mGluR5) contributes to pain and c-fos expression in a rat model of inflammatory pain. Pain 158, 705–716. 10.1097/j.pain.0000000000000823 28030475

[B76] Volkow N. D. McLellan T. A. Cotto J. H. Karithanom M. Weiss S. R. B. (2011). Characteristics of opioid prescriptions in 2009. JAMA 305, 1299–1301. 10.1001/jama.2011.401 21467282 PMC3187622

[B77] Whelton A. (2000). Renal and related cardiovascular effects of conventional and COX-2-specific NSAIDs and non-NSAID analgesics. Am. J. Ther. 7, 63–74. 10.1097/00045391-200007020-00004 11319575

[B78] Yarwood R. E. Imlach W. L. Lieu T. Veldhuis N. A. Jensen D. D. Klein Herenbrink C.. (2017). Endosomal signaling of the receptor for calcitonin gene-related peptide mediates pain transmission. Proc. Natl. Acad. Sci. U S A 114, 12309–12314. 10.1073/pnas.1706656114 29087309 PMC5699040

[B79] Zhao P. Lieu T. Barlow N. Metcalf M. Veldhuis N. A. Jensen D. D.. (2014). Cathepsin S causes inflammatory pain *via* biased agonism of PAR2 and TRPV4. J. Biol. Chem. 289, 27215–27234. 10.1074/jbc.M114.599712 25118282 PMC4175355

[B80] Zhao P. Lieu T. Barlow N. Sostegni S. Haerteis S. Korbmacher C.. (2015). Neutrophil elastase activates protease-activated receptor-2 (PAR2) and transient receptor potential vanilloid 4 (TRPV4) to cause inflammation and pain. J. Biol. Chem. 290, 13875–13887. 10.1074/jbc.M115.642736 25878251 PMC4447962

